# Odoriferous Feet

**Published:** 1861-01

**Authors:** 


**ODORIFEROUS FEET.**—You can smell some people a mile off—be the same more or less. That an odor issues from every person peculiar to himself, is proven by the fact that the dog can find his master although out of sight; but this emanation from the body is so ethereal generally, that the human sense of smell can not distinguish it. In very rare instances the calamity may be inherited, or may arise from a scrofulous constitution. At the same time it is true, that in almost every case, bad-smelling feet, or person, arises from old perspiration in a decaying condition. There is no special odor to the perspiration from the hands. It is because they are constantly exposed to the air and are frequently washed and ventilated; and so with the face. It is from the feet always covered; from the arm-pits seldom washed; and from the groins always in a perspiring condition, that fetid odors come. The remedy then is the plentiful and frequent application of soap and hot water, twice a day, as long as needed. This may not avail sometimes; especially with men, for many keep their boots on the whole day; the perspiration of the feet condenses on them, decomposes, and the gas given out is absorbed by the leather, and remains permanently. In such cases not only is the strictest personal cleanliness necessary, the toes and nails being very particularly attended to, but shoes should be worn to allow of a more free escape of gases; they should be changed every day; and when not on the feet, should be exposed to the out-door air, so as to have a most thorough ventilation.

"Aqua Ammonia" (Hartshorn water) is used by some for the removal of unpleasant personal odors; but it has one of its own scarcely more agreeable, and perhaps it acts only by having a stronger smell. The most efficient plan is attention to the strictest cleanliness and the use of shoes, as above, and if, in addition, a high state of general health is maintained by temperance and exercise out of doors several hours daily, the most inveterate fetors will seldom fail of removal.

